# Genetic Testing and Counselling in Hypertrophic Cardiomyopathy: Frequently Asked Questions

**DOI:** 10.3390/jcm12072489

**Published:** 2023-03-24

**Authors:** Francesca Girolami, Alessia Gozzini, Eszter Dalma Pálinkás, Adelaide Ballerini, Alessia Tomberli, Katia Baldini, Alberto Marchi, Mattia Zampieri, Silvia Passantino, Giulio Porcedda, Giovanni Battista Calabri, Elena Bennati, Gaia Spaziani, Lia Crotti, Franco Cecchi, Silvia Favilli, Iacopo Olivotto

**Affiliations:** 1 Pediatric Cardiology Unit, Meyer Children’s Hospital IRCCS, 50139 Florence, Italy; 2 Doctoral School of Clinical Medicine, University of Szeged, 6720 Szeged, Hungary; 3 Cardiomyopathy Unit, Careggi University Hospital, 50134 Florence, Italy; 4 Department of Cardiovascular, Neural and Metabolic Sciences, San Luca Hospital, Istituto Auxologico Italiano, IRCCS, 20100 Milan, Italy; 5 Department of Medicine and Surgery, University Milano Bicocca, 20126 Milan, Italy; 6 Department of Experimental and Clinical Medicine, University of Florence, 50121 Florence, Italy

**Keywords:** hypertrophic cardiomyopathy, genetic testing, genetic counselling, cascade testing, multidisciplinary team, next-generation sequencing

## Abstract

Genetic counselling and genetic testing in hypertrophic cardiomyopathy (HCM) represent an integral part of the diagnostic algorithm to confirm the diagnosis, distinguish it from phenocopies, and suggest tailored therapeutic intervention strategies. Additionally, they enable cascade genetic testing in the family. With the implementation of Next Generation Sequencing technologies (NGS), the interpretation of genetic data has become more complex. In this regard, cardiologists play a central role, aiding geneticists to correctly evaluate the pathogenicity of the identified genetic alterations. In the ideal setting, geneticists and cardiologists must work side by side to diagnose HCM as well as convey the correct information to patients in response to their many questions and concerns. After a brief overview of the role of genetics in the diagnosis of HCM, we present and discuss the frequently asked questions by HCM patients throughout our 20-year genetic counselling experience. Appropriate communication between the team and the families is key to the goal of delivering the full potential of genetic testing to our patients.

## 1. Introduction

Hypertrophic Cardiomyopathy (HCM) is a genetic heart condition characterized by the presence of asymmetric hypertrophy of the left ventricle (LVH) that is not solely explained by abnormal loading conditions [1,2,3,4,5]. However, in the latest AHA/ACC HCM guidelines, the term is applied only to patients that have a sarcomeric gene variant or a negative gene test in the absence of another cardiac, systemic, or metabolic disease [5]. In the 1990s, an *MYH7* (β myosin heavy chain) variant was identified in a large Canadian family with four generations of HCM-affected subjects [6,7]. Over the past 30 years, the complexity of the genetic basis of HCM has been increasingly elucidated. Today HCM is considered the most common genetic cardiac disease, transmitted in an autosomal dominant manner. HCM has a prevalence of roughly 1:500 in adults, thus affecting an estimated 1 million individuals in Europe [8]. Mutation penetrance varies widely and may be significantly delayed [9]. HCM is known to be associated with over ~1500 mutations, described in about 40 genes, especially in genes encoding for cardiac sarcomeric proteins. HCM has been designed as the “disease of the sarcomere” [10]. *MYH7* and *MYBPC3* (cardiac myosin-binding protein C) are the two most commonly mutated sarcomeric genes, and they account for about 40% of diagnoses. In addition, *MYL2* (regulatory myosin light chain), *MYL3* (essential myosin light chain), *TPM1* (α tropomyosin), *TNNT2* (cardiac troponin T), *TNNI3* (cardiac troponin I), and *ACTC1* (cardiac actin) represent the “core disease” genes which are considered essential for establishing a reliable and accurate molecular diagnosis. Rare mutations have been found in additional sarcomere genes or genes coding for proteins from the nearby Z-disc, such as *MYH6* (α myosin heavy chain), *CSRP3* (muscle LIM protein), *TCAP* (telethonin), and genes implicated in calcium homeostasis pathways, such as *VCL* (vinculin) and *JPH2* (junctophilin 2) [11,12,13,14]. A small number of hypertrophic patients exhibit Fabry syndrome (*GLA* gene), Danon disease (*LAMP2* gene), cardiac amyloidosis (*TTR* gene), Wolff—Parkinson—White syndrome (*PRKAG2* gene), and mitochondrial cardiomyopathies [15,16,17,18]. These phenocopies are different disease entities with distinct heredity, pathogenesis, natural history, extra-cardiac characteristics, and therapy. Moreover, HCM phenotype may be associated with malformation syndromes, mainly RASophaties, a heterogeneous group of diseases caused by mutations in genes of RAS-MAPK cascade, including *PTPN11*, *BRAF*, *RAF1*, *SOS1*, *HRAS*, and *KRAS* [14]. For these diseases, the genetic test, in conjunction with a detailed diagnostic examination of the patient and their family, is a critical tool for the diagnosis and proper treatment [19].

Currently, with Next Generation Sequencing (NGS) technologies, it is possible to identify a responsible mutation in 30–60% of HCM patients. However, it is true that the yield of positive genetic testing is higher in the case of positive family history of HCM and is lower in sporadic cases [5,20]. The genetic cause remains unknown and may be polygenic in the rest of the cases, together with some environmental factors, such as obesity, arterial hypertension, and intense sports activity [14,20]. About 5–10% of patients have been reported to have complex genotypes carrying multiple (two or more rarely three) mutations in sarcomere genes; this condition is usually associated with a poorer prognosis [21]. Since 2005, NGS technologies have enabled a breakthrough rise in high-throughput sequencing capabilities by analyzing multigene panels, whole-exome (WES), and whole-genome sequencing (WGS). The latter is mostly aimed at discovering novel disease-causing genes and is mostly used for research purposes. In selected cases, for example, in children with HCM, the WES analysis in a trio (together with the parent’s DNA sequencing) should be performed [22]. The NGS strategy is to sequence millions of short-DNA fragments in massively parallel arrays, then realign and map the short reads back to the reference genome [23]. The accurate interpretation of data sequencing is the weakness of the NGS strategy. It may be very difficult to predict if a DNA variant identified in a patient is truly disease-causing (pathogenic or likely pathogenic), a benign polymorphism (negative test) or a rare variant with unclear clinical significance and not predicted to cause HCM (variant of uncertain significance, VUS) [24]. In this last case, the genetic test’s result is not clinically actionable and may be considered one limitation of the genetic study. Private and public cardiogenetic laboratories often use commercial panels for genetic tests. The panels are typically designed to contain genes having a definitive or moderate association with the disorders; the panels must be assessed on a regular basis and may potentially be expanded if new genes are identified [10,20,25].

## 2. Genetic Counselling

**Figure 1:**
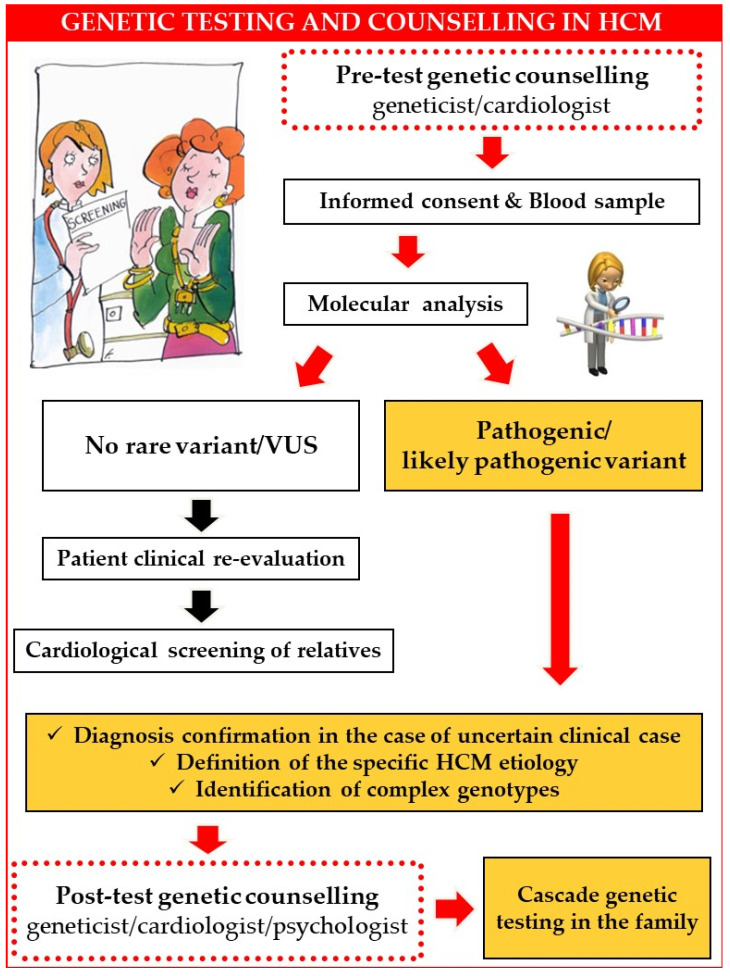
Genetic testing strategy in HCM. Abbreviations: HCM: hypertrophic cardiomyopathy; VUS: variant of uncertain significance.

Genetic counselling represents an essential part of genetic testing and is recommended for all patients with HCM who choose to undergo genetic testing [5]. The main objective of counselling is informing patients and families about the genetic aspects of their disease, the advantages and the limitations of the test and the possibility of transmitting the disease to their relatives [26,27,28,29]. It is also critical to help ensure that patients and families have realistic expectations about the benefits of genetic testing. Counselling can be divided into pre-test and post-test counselling (Figure 1). According to the latest guidelines on HCM, pre-and post-test genetic counselling should be performed by a trained genetic counsellor or by other experts in the genetics of cardiovascular diseases (Class 1, level of evidence B) [5]. Due to technological advances and new recommendations, the number of genetic testing requests is increasing rapidly, which may expose cardiologists to more often facing similar questions about the implication of genetic testing results. Therefore, in this article, we collected and discussed the frequently asked questions by our HCM patients to help cardiologists manage genetic testing-related scenarios more easily and confidently.

***Pre-test counselling***. During pre-test counselling, the patient should be informed about the specific type of test that will be performed on the DNA sample (for example, which are the genes studied and by which technologies), the possible test results, the potential clinical benefit, and the test turn-around time. Pre-test counselling is of paramount importance to identify other clinically affected family members, patterns of disease transmission, consanguinity within the family, and history of sudden cardiac death in a relative. Moreover, it helps to assist decision-making by fully informing patients [5,23]. A three generations pedigree of the family, written informed consent and a blood sample are collected during this interview.

***Post-test counselling***. Test results should be communicated during post-test genetic counselling. Post-test genetic counselling should involve skilled healthcare professionals working in multidisciplinary teams to assist patients in understanding and handling the psychological, social, professional, ethical, and legal consequences of test results. During post-test genetic counselling, the patient becomes aware of the impact of test results on himself/herself and his/her relatives. Importantly, the patient is informed about the clinical implications and the possibility of performing cascade testing screening on other family members (predictive genetic testing in relatives). In young patients, the risk of transmission when planning future pregnancies should be discussed [5].

## 3. Patients’ Questions, Real-World Answers

Interpreting the clinical significance of genetic variants is a complex process in which the role of clinicians is key by providing evidence that supports the variants’ classification, both in the proband and in family members [23]. Cardiologists need to be able to answer questions from patients with genetic heart diseases, collaborating closely with the geneticist. In this article, we present and discuss the frequently asked questions by HCM patients over our 20-year genetic counselling experience.

***Why is it useful to undergo a genetic test for me*?** Genetic testing in HCM adult patients remains critical for the identification of at-risk asymptomatic relatives. In addition, it provides a definitive molecular diagnosis and allows the identification of phenocopies (for example Anderson-Fabry disease, *TTR* amyloidosis, Danon disease, or *PRKAG2* cardiac glycogenosis). Conversely, the impact on risk stratification and the prognosis is limited as genotype status alone cannot predict patient-specific outcomes. However, it is important to note that patients with pathogenic or likely pathogenic sarcomere mutations had a greater risk for adverse outcomes compared with patients without mutations [5,10,11,21].

***Will the identified DNA mutation predict the course and outcome of my disease?*** Because HCM is a genetic disease with incomplete penetrance and variable expressivity, the possibility of associating mutations with a specific disease course is limited. Some genes or specific mutations have been linked to a high rate of sudden mortality and adverse remodeling (e.g., *TNNT2* mutations; *MYH7* p.Arg403Gln), a low rate of LVH (*TNNT2*), or delayed cardiac expression (*MYBPC3*). Additionally, patients with complex genotypes are more likely to have severe phenotypes or early disease manifestations [21]. Yet, no clear and consistent associations have been discovered for the majority of mutations to date [20,22,23,30,31,32].

***Is it possible that I will never develop the disease, even if I have inherited the familial mutation?*** HCM is known to be a disease with incomplete penetrance and an intra-family variability of phenotype expression. Hence, the identification of a sarcomeric mutation (genotype positive) in an otherwise healthy individual (phenotype negative) does not necessarily mean that the subject will develop the disease but does indicate an increased risk of developing it. Although HCM may rarely be present in neonates and children, usually, the mean age of onset in HCM ranges from the third to the fifth decade of life. However, it has been reported that in a proportion of cases, it may remain latent or be identified even after age 60 [33].

***If my genetic mutation gets identified, will I have targeted therapies?*** Although experimental studies hold hope for the future, no etiological/preventive therapies are available today. Nonetheless, the identification of a pathogenic mutation may help the cardiologist make tailored therapeutic choices and distinguish rare mimics such as Fabry disease, Danon disease, amyloidosis caused by *TTR* mutation, or atypical glycogenosis due to mutations in the *PRKAG2* gene. Emerging, targeted therapies, including gene therapy for HCM patients, are now opening new perspectives [24,34,35,36,37,38].

***In the case of an uncertain clinical diagnosis, can the identification of a genetic mutation definitively help to clarify it?*** In the case of inconclusive clinical findings, the identification of a pathogenic/likely pathogenic HCM rare variant can confirm the diagnosis [39].

***My echocardiography is normal, but my genetic test is positive. Should I stop the physical activity?*** In general, for most patients with HCM, mild- to moderate-intensity recreational exercise is beneficial and is recommended to improve cardiorespiratory fitness, quality of life, and overall health. For phenotype-negative individuals, due to their low risk of sudden cardiac death (SCD), there is no restriction from competitive sports unless the family history indicates a high risk for SCD. However, serial echocardiography is recommended to assess for any phenotype development at periodic intervals depending on age [1,5,40].

***Can a positive genetic test influence my insurance policies?*** Genetic data are strictly personal and protected by different regulations from country to country. In the USA, The Genetic Information Non-discrimination Act (GINA) protects individuals against discrimination based on their genetic information in health coverage and employment. Italy also has these types of laws (Authorization No. 8/2016—Garante Privacy). For this reason, it is not required to declare to have carried out the genetic test, either in the working environment or during the stipulation of life insurance [41].

***How likely is it that my children will inherit the mutation?*** Sarcomeric HCM is inherited in an autosomal dominant manner, which means that both female and male children have a 50% probability of carrying the disease-causing variant, although variable penetrance can result in differences in the onset and severity of clinical manifestations [5].

***I know genetic test needs a long time to be performed. However, once a mutation has been identified, is it going to take that much time to test my family?*** Once the mutation is identified in the first patient of the family, we can easily look for it in the other family members. We already know which “word” is misspelled and on which “page” it is located. Therefore, the predictive test takes only a few days [42].

***Since I have HCM and a causative mutation has been found, is it possible to perform the genetic test on my five-year-old son/daughter, even if he/she does not show any sign of the disease?*** Performing a genetic test on an unaffected child is generally not recommended [43]. The fundamental justification behind this is the lack of knowledge on the severity, age of onset, and phenotypic manifestations associated with HCM, as well as the inability to stop its progression. For these reasons, the medical benefit of a pre-symptomatic diagnosis of HCM is questionable, and since the request is made by the parents, the child is often not mature enough to understand the consequences of the test and make any decision. In our opinion, it is of utmost importance to balance benefits and harms, case by case, and keep in mind that he/she should be free to choose by himself to know whether he/she has inherited the mutation. Therefore, in the case of an unaffected child, a genetic test should be carried out after 10–12 years of age. However, in certain situations, such as a possible competitive sports career or a family history of malignant early-onset disease, it may be performed earlier [22,44].

***After the identification of the mutation in my husband, is it possible to perform a prenatal test on our child?*** The decision to pursue a prenatal diagnosis is a personal one. Through invasive prenatal diagnosis (by amniocentesis or chorionic villus sample), it is possible to obtain the fetus’s DNA and proceed with searching for the known mutation [5,23,26]. Nonetheless, in our opinion, it is not appropriate to carry out this type of test due to the above-mentioned reasons: the uncertain age of onset of symptoms, the severity of symptoms, the risk of complications, etc. Clinicians cannot precisely anticipate the clinical course of an individual with a mutation since extremely different manifestations of HCM can occur in family members carrying the same mutation. Both incomplete penetrance and variable expressivity are probably due to a combination of genetic, environmental, and lifestyle factors. In our experience, none of the couples persisted to request prenatal testing after the first steps of the multidisciplinary shared decision-making process.

***If the mutation is not identified, does it mean that my cardiomyopathy does not have a genetic base?*** The available genetic test in the cardiogenetic laboratories involves the analysis of a panel of genes known to be associated with HCM. The probability of identifying a disease-causing mutation in these genes is about 40–60% (test sensitivity). It is believed that both familial and isolated HCM forms are genetically determined. Therefore, even if no causative variant gets identified, a genetic basis cannot be excluded [5]. In this scenario, the causative variant could lie in modulator or unknown genes or in DNA regions that we are not able to analyze yet. For these reasons, both the HCM patients with negative genetic tests and their relatives should undergo regular cardiological follow-ups [5].

***What does “Inconclusive genetic test result” mean?*** It means that a rare variant was identified in the patient’s DNA, called a variant of uncertain significance (VUS). It is unclear whether this variant is associated with the health condition or not. Due to their rarity in the population, only a limited amount of information is available on VUS. Thus, when a VUS gets identified, the test result is ambiguous and inconclusive at a clinical level. For this reason, a VUS should not be used to establish a genetic diagnosis and guide cascade family screening. Future knowledge or scientific reports may ameliorate our understanding of the VUS and help to re-evaluate them. The identification of a VUS, even if it represents one of the three possible results of a genetic test (1, positive: pathogenic/likely pathogenic causative variant; 2, rare VUS; 3, no identified rare variant) can be considered a limitation of genetic testing [24,45,46].

In Table 1, we have collected some pieces of information for cardiologists who deem it appropriate to have the patient undergo genetic testing. We consider these recommendations necessary to avoid setting unrealistic expectations and to ensure that it is consistent with the information provided during genetic counselling.

## 4. Conclusions

For cardiologists to achieve confidence in ordering genetic testing for HCM and counselling, they must be able to answer the major questions of their patients. Continuing close collaboration with geneticists is the key to success.

## Figures and Tables

**Table 1:** Recommendations for the cardiologist on genetic testing in HCM.

***Genetic Testing in HCM: Tips for the Cardiologist***
***It is important to inform the patient that genetic testing:***
Usually needs a long time (up to three months) before results are ready.
Has a chance of finding a causative mutation ranging from 30 to 60%, depending on the cardiomyopathy and its presence in other family members.
Can be extended to other family members **ONLY** in the presence of a pathogenic/likely pathogenic variant in the proband.
***It is undesirable to suggest:***
Performing the test as a matter of urgency.
Waiting for the test result to start treatment.
Submitting the entire family to genetic testing before determining the proband’s mutation.
Advising a prenatal diagnosis before genetic counselling.
Waiting for test results to decide on physical and sport activity.
Performing genetic testing for individuals in the family without phenotypic evidence of HCM, including relatives of sudden cardiac death victims.

## Data Availability

No new data were created or analyzed in this study. Data sharing is not applicable to this article.
